# Gamma-oryzanol supplementation enhances laying performance, egg quality, and ovarian follicular development in Japanese quails

**DOI:** 10.14202/vetworld.2025.2563-2572

**Published:** 2025-09-06

**Authors:** Kunlayaphat Wuthijaree, Wilasinee Inyawilert, Pattaraporn Tatsapong, Amornrat Wanangkarn, Anurak Khieokhajonkhet, Chompunut Lumsangkul

**Affiliations:** 1 Department of Agricultural Science, Faculty of Agriculture Natural Resources and Environment, Naresuan University, Phitsanulok, Thailand; 2 Department of Animal Science, National Chung Hsing University, Taichung, Taiwan

**Keywords:** antioxidant feed additive, egg quality, follicular development, gamma-oryzanol, Japanese quails, laying performance

## Abstract

**Background and Aim:**

Gamma-oryzanol, a bioactive compound derived from rice bran, is recognized for its antioxidant and metabolic regulatory properties. This study evaluated the effects of dietary gamma-oryzanol supplementation on laying performance, egg quality, blood health, and follicular development in Japanese quails (*Coturnix japonica*).

**Materials and Methods:**

A total of 216 12-week-old laying quails were randomly assigned to six groups receiving ­gamma-oryzanol at 0 (control), 0.1, 0.2, 0.3, 0.4, and 0.5 g/kg of diet for 7 weeks. Feed intake, egg production, feed conversion ratio, egg quality traits, hematological and biochemical parameters, and ovarian follicular characteristics were measured. Statistical analysis was conducted using one-way analysis of variance and Duncan’s *post hoc* test.

**Results:**

Gamma-oryzanol at 0.2 g/kg significantly increased egg production (98.69%) compared to the control (86.80%). Supplementation at 0.3–0.5 g/kg improved eggshell thickness and yolk index (p < 0.05). Red blood cell counts were significantly higher at 0.4 g/kg, indicating enhanced erythropoiesis. Other hematological and biochemical parameters remained unchanged. A significant increase in small white follicle count and F3 follicle diameter was observed at 0.5 g/kg, indicating improved follicular development.

**Conclusion:**

Gamma-oryzanol supplementation at 0.2–0.5 g/kg enhanced laying performance, egg quality, and ovarian follicle development without adverse hematological or biochemical effects. The compound shows potential as a safe and functional feed additive to support productivity and reproductive efficiency in commercial quail production.

## INTRODUCTION

Gamma-oryzanol, a bioactive component derived from rice bran, has attracted interest as a natural feed additive due to its potent antioxidant and health-promoting properties [1]. It consists of ferulic acid esters of phytosterols and triterpenoids, which contribute to its free radical scavenging ability and lipid peroxidation prevention [2]. This compound has demonstrated anti-inflammatory and hypocholesterolemic effects alongside its antioxidant activity in various animal models and *in vitro* studies [1]. These attributes make gamma-oryzanol a promising nutraceutical in poultry nutrition, with the potential to enhance bird performance, product quality, and overall health. However, its use in poultry diets, particularly in laying Japanese quails, remains underexplored, and further investigation is required to determine its efficacy and optimal dosage.

A recent study by Abeyrathna *et al*. [3] reported that dietary inclusion of rice bran above 20% negatively affected laying performance in Japanese quails, especially without phytase supplementation. In contrast, supplementation with extracted rice bran oil (RBO) improved egg production in laying hens without affecting feed intake [4]. Gamma-oryzanol supplementation may influence egg quality parameters and the birds’ physiological health profile beyond performance metrics. For example, a study on quails fed rice bran – a rich source of gamma-oryzanol – reported a marked reduction in egg yolk lipids and cholesterol compared with the control group [5]. This cholesterol-lowering effect is consistent with the known hypocholesterolemic action of gamma-oryzanol in other species [1, 5].

Enhancing the lipid profile of eggs benefits quail physiology and consumer health, especially given the demand for low-cholesterol eggs. In the same quail study, blood biochemical indices were positively altered – birds receiving gamma-oryzanol through rice bran had lower serum triglycerides and low-density lipoprotein-cholesterol, along with higher high-density lipoprotein-cholesterol, relative to unsupplemented birds [5]. This improved blood lipid profile suggests an overall cardiovascular health benefit, presumably linked to the sterolin structure of gamma-oryzanol, which can interfere with cholesterol absorption or synthesis [5].

Moreover, the antioxidant role of gamma-oryzanol was evidenced by a marked increase in the total antioxidant capacity (TAC) of quails in plasma, by approximately 33%–66% in the supplemented groups [5]. Such enhancement of antioxidant status indicates that gamma-oryzanol can reduce oxidative stress in laying birds, which may underlie many of the observed improvements in performance and egg quality. Available studies indicate that gamma-oryzanol does not negatively impact hematological parameters such as red or white blood cell counts, suggesting that it is physiologically safe for avian species. However, specific studies focusing on hematology are sparse and warrant further investigation.

In addition, dietary supplementation with RBO has shown positive outcomes in laying performance and reduced total serum cholesterol in layer chickens [6]. In broilers, supplementation with RBO, rich in gamma-oryzanol, has led to improved growth performance and favorable blood lipid profiles [7]. Gamma-oryzanol may also influence reproductive physiology, particularly ovarian follicular development, in laying quails. The ovaries of laying hens and quails contain follicles at different maturation stages, and the number and health of these follicles directly affect egg production rate and consistency.

Other poultry studies have shown that antioxidant supplementation benefits ovarian function. For example, dietary canthaxanthin (a carotenoid antioxidant) improved ovarian morphology and increased the number of developing follicles in hens, ultimately boosting their laying rate [8]. This suggests that reducing oxidative stress in the ovary can promote follicular development and reduce follicle atresia, thereby enhancing reproductive performance [8]. Given the antioxidant capacity of gamma-oryzanol, it is plausible that it could have similar positive effects on the ovaries of quails, such as supporting the growth of ovarian follicles or balancing reproductive hormones.

However, to date, there is a clear gap in research regarding how gamma-oryzanol affects the follicle characteristics in laying quails. In the past few years, no peer-reviewed studies have specifically examined follicle numbers, sizes, or ovarian histology in quails supplemented with gamma-oryzanol, leaving this potential benefit largely hypothetical. This knowledge gap emphasizes the need for targeted studies to determine whether gamma-oryzanol improves reproductive traits, such as follicular growth and ovulation, beyond its known effects on performance.

Gamma-oryzanol is emerging as a promising natural feed additive in poultry production owing to its antioxidant and cholesterol-lowering properties. Recent evidence (primarily from chickens) indicates that it can enhance laying performance, feed efficiency, and egg quality and improve blood lipid profiles while bolstering antioxidant status [5].

Despite these promising effects, the influence of gamma-oryzanol on egg quality traits (including shell strength and yolk composition), hematological and biochemical health indices, and ovarian follicle dynamics in quails remains insufficiently explored. Specifically, its effects on follicle number, development stages, and egg-associated physiological parameters in laying Japanese quails are poorly documented. Moreover, there is limited evidence on its safe inclusion levels and long-term impact on reproductive health in this species. These gaps restrict the optimization of its use in quail production systems.

Therefore, this study aimed to evaluate the effect of gamma-oryzanol supplementation on laying performance, egg quality, blood parameters, and follicular development in Japanese quails, providing insight into its physiological benefits. The findings are expected to clarify the role of gamma-oryzanol as a functional feed additive and its potential to enhance the sustainability and productivity of quail egg production.

## MATERIALS AND METHODS

### Ethical approval

The study was conducted in accordance with animal welfare guidelines and approved by the Institutional Animal Care and Use Committee (IACUC) of Naresuan University (Protocol No. NU-AG670904; Approval No. 6801003).

### Study period and location

This study was conducted from October to November 2024 at the Faculty of Agriculture, Natural Resources and Environment, Naresuan University, Phitsanulok, Thailand.

### Experimental birds and housing

A total of 216 Japanese laying quails (*Coturnix japonica*) aged 12 weeks were randomly assigned to six treatment groups in a completely randomized design. Each group consisted of six replicates, with six birds per replicate housed in individual cages. The study period spanned from 12 to 19 weeks of age. In a cage measuring 50 × 30 × 50 cm, there were 6 birds (250 cm²/quail). Each cage was equipped with a nipple drinker and a trough feeder. The birds were reared under natural environmental conditions, with ambient temperatures ranging from 25.4°C (morning) to 34.3°C (evening), and relative humidity maintained between 62% and 70%. Water and diet were available at all times during the study. Vaccinations and health management protocols were implemented under veterinary supervision according to age-specific schedules.

### Diet formulation and treatment groups

In the first group (control), the birds were fed the basal diet (BD) without gamma-oryzanol supplementation. The second to sixth groups received the BD supplemented with gamma-oryzanol at 0.1, 0.2, 0.3, 0.4, and 0.5 g/kg diet, respectively. Gamma-oryzanol powder (97% purity, rice bran extract) was obtained from Chanjao Longevity Co., Ltd., Bangkok, Thailand. The BD was formulated to contain 22.25% crude protein, 2,948 kcal/kg metabolizable energy, 3.34% calcium, and 0.5% phosphorus, following the National Research Council (NRC) recommendations [9], and fed as a mash, as shown in Table 1.

**Table 1:** Ingredients and nutrient contents of experimental diets used by laying quails.

Ingredient	Percentage
Corn	43
Soybean meal (43% CP)	38
Rice barn	6
Fish meal (60% CP)	3.5
Limestone	7.4
Dicalcium phosphate	1
DL-Methionine	0.3
Salt	0.3
Premix*	0.5
Calculated composition**	
ME (Kcal/kg)	2948
Crude protein	22.25
Calcium	3.34
Nonphytate phosphorus	0.5
Lysine	1.27
Total sulfur amino acids	0.77

* Premix provides per 1 kg of diet: Mn: 80 mg, Fe: 60 mg, Cu: 5 mg; I: 1 mg, Se: 0.15 mg, Vitamin A: 8.800 IU, Vitamin D3: 2.200 IU, Vitamin E: 11 mg, Vitamin K3: 2.5 mg, Nicotinic acid: 44 mg, Calcium-D-pantothenate: 8.8 mg, Riboflavin: 4.4 mg, Thiamine: 2.5 mg, Vitamin B12: 6.6 mg, Folic acid: 1 mg, Biotin: 0.11 mg, Choline: 220 mg.

** Calculated according to the guidelines of the National Research Council [9]

### Performance evaluation

The body weights of the participants were recorded at the beginning and end of the study using a precision balance with 0.01 g precision, and the body weight (g) change was calculated from these data.

Egg production (%) = (Total number of eggs in the period [number]/Number of quails in the group [number]) × 100.

Egg weights (g) were determined by weighing all eggs collected from each replicate in the last 2 days of each 7-day period. The egg mass (g) [10] was calculated by dividing the percentage of egg yields for each week by the average egg weight and then by 100. The quails were weighed and fed in groups, and their daily feed intake (g) was calculated.

The feed conversion ratio (FCR) was calculated as the ratio of daily feed intake to the corresponding egg mass (g feed/g egg mass).

### Egg quality assessment

The egg quality assessment included both internal and external parameters. Three eggs from each treatment duplicate were used to measure egg components weekly and were used as the average of all trials.

According to Cufadar *et al*. [11], eggshell quality characteristics were determined in a total of 3 eggs from each replicate on the past 2 days of each 7-day period (a total of 108 eggs were examined in each treatment group during the trial period; 864 eggs were examined in all treatment groups during the 8-week trial).

The eggshell weight (with membrane) was determined after the eggs were broken and their contents were separated, cleaned thoroughly, dried at room temperature (27ºC), and weighed using a precision digital scale.

The eggshell ratio was calculated by dividing the eggshell weight by the egg weight.

Eggshell thickness (excluding the shell membrane) was determined by taking the average of the measurements made from 3 points at the air cell, equator, and sharp end of the eggs with a digital micrometer (Mitutoyo-Series 102, Misumi (Thailand) Co., Ltd.).

The yolk index was determined after eliminating the yolk from the albumen.

Egg yolk index = (yolk height/yolk diameter) × 100 [12]

Albumen index (%) = (albumen height (mm)/[(albumen length (mm) + albumen width (mm)]/2) × 100

Haugh unit = 100 × log (albumen height + 7.57 − 1.7 × egg weight^0.37^) [13].

Yolk color was evaluated using the DSM-Firmenich YolkFan (Switzerland), with values assigned based on visual color matching.

### Hematological and biochemical analysis of blood

Blood samples from 36 birds (6 birds/treatment) were collected in the morning from the wing veins at the end of the trials. Blood samples were collected in an ethylenediaminetetraacetic acid (EDTA) tube and a serum separation tube. After centrifugation at 3000 rpm for 10 min, serum was collected and stored at −20°C until analysis.

Biochemical parameters, including total protein (TP), albumin (ALB), globulin (GLOB), creatinine, blood urea nitrogen (BUN), alanine aminotransferase (ALT), and aspartate aminotransferase (AST), were analyzed using an automated biochemical analyzer (Hitachi 7180, Tokyo, Japan). Blood samples collected in EDTA tubes were determined on the day of sampling. The total white blood cell (WBC) and red blood cell (RBC) counts were determined using a Neubauer hemocytometer with Hayem solution. The hemoglobin (Hb) concentration was determined using the cyanmethemoglobin method at 540 nm.

### Ovarian evaluation and follicle characterization

At the end of the experiment, 6 quails per treatment (one bird from each replicate) were weighed and slaughtered. Ovaries were collected and weighed. The ovarian indices (ovarian weight [g]/body weight [g] × 100%) were calculated [14].

Follicle types were classified and counted as follows:

- Primary follicles (>8 mm)
- Small yellow follicles (SYF) (4–8 mm)
- Large white follicles (LWF) (2–4 mm)
- Small white follicles (SWF) (≤1 mm) [15].

### Statistical analysis

All the data generated in this study were subjected to one-way analysis of variance (ANOVA), which ensures that there is no bias with respect to data normality and equality of variance assumptions. Data were analyzed using a one-way ANOVA followed by Duncan’s multiple range tests to determine significant differences (p < 0.05). Data were analyzed using the Statistical Package for the Social Sciences software version 17.0 (IBM Corp., Armonk, NY, USA). Statistical differences are presented as mean ± standard deviation (SD) in the tables. Results were deemed statistically significant if treatment means had different superscript letters in the same column (e.g., a, b, and c), indicating differences at (p < 0.05). Data interpretation followed the established standards for poultry performance metrics.

## RESULTS

### Laying performance

There were no significant differences in the initial body weight, final body weight, body weight change, feed intake, or egg weight of laying quails among the treatment groups (p > 0.05) (Table 2). Egg production was significantly increased with gamma-oryzanol supplementation (p < 0.05), peaking at 98.69% in the 0.2 g/kg diet group, which was significantly higher than the control and 1 g/kg groups. Quails supplemented with 0.3–0.5 g/kg diet also showed elevated egg production (93.78%–95.66%), although differences from the 0.2 g/kg group were not statistically significant. Egg mass tended to increase in the 0.2 g/kg diet group (11.26 g/day), although the difference was not statistically significant. A trend toward improved FCR was observed with higher gamma-oryzanol supplementation. Nevertheless, the disparities were not statistically significant.

**Table 2:** Body weight, feed intake, and laying performance of laying quails fed with gamma-oryzanol supplementation (n = 36 birds/experimental group).

Performance parameters	Gamma oryzanol level (g/kg diet)						p-value

	0	0.1	0.2	0.3	0.4	0.5	
IBW, g	169.66 ± 20.62	174.77 ± 9.25	167.22 ± 14.92	157.04 ± 20.05	147.27 ± 23.84	157.36 ± 26.35	0.059
FBW, g	195.88 ± 17.88	187.50 ± 18.47	188.86 ± 21.15	191.44 ± 29.52	185.82 ± 14.04	196.59 ± 20.13	0.837
BWC, g	26.22 ± 21.22	12.73 ± 13.09	21.64 ± 22.44	34.40 ± 25.29	38.56 ± 25.01	39.23 ± 26.89	0.110
FI, g/bird/day	26.64 ± 1.10	26.15 ± 1.99	27.90 ± 1.27	26.92 ± 2.38	25.80 ± 1.44	26.64 ± 0.92	0.303
EP, %	86.80 ± 9.61^b^	87.39 ± 9.41^b^	98.69 ± 1.80^a^	93.78 ± 7.61^ab^	95.57 ± 7.49^ab^	95.66 ± 6.21^ab^	0.035
EW, g	11.53 ± 0.35	11.30 ± 0.34	11.41 ± 0.32	11.51 ± 0.52	11.15 ± 0.42	11.51 ± 0.19	0.353
EM, g/day	10.02 ± 1.26	9.88 ± 1.14	11.26 ± 0.34	10.78 ± 0.89	10.65 ± 0.94	11.01 ± 0.75	0.070
FCR, FI/EM	2.69 ± 0.28	2.67 ± 0.20	2.49 ± 0.11	2.51 ± 0.23	2.44 ± 0.16	2.44 ± 0.16	0.083

IBW = Initial body weight, FBW = Final body weight, BWC = Body weight change, EP = Egg production, FI = Feed Intake, EW = Egg weight, EM = Egg mass FCR = Feed conversion ratio. ^a,b^Means in the same row within each classification bearing different letters are significantly (p < 0.05) different

### Egg quality

No significant effects were observed on the egg shape index, albumin percentage, yolk percentage, or shell percentage across the treatment groups (p > 0.05) (Table 3). The shell thickness was significantly influenced by gamma-oryzanol supplementation (p < 0.05). The greatest shell thickness (0.27 mm) was found in the 0.3–0.5 g/kg supplementation groups, whereas the 0.1 g/kg and 0.2 g/kg diet groups had significantly lower values (0.26 mm). Furthermore, there were no statistically significant differences in the internal egg quality, including the albumin index and Haugh unit score, between the treatments (p > 0.05). Interestingly, yolk index values were significantly increased in the 0.3–0.5 g/kg diet groups (p < 0.05).

**Table 3:** Average egg quality of laying quails fed with gamma-oryzanol supplementation (n = 144 eggs/experimental group).

Egg quality parameters	Gamma oryzanol level (g/kg diet)						p-value

	0	0.1	0.2	0.3	0.4	0.5	
Egg shape index (%)	76.98 ± 2.62	75.34 ± 3.28	77.61 ± 6.04	77.12 ± 3.03	76.21 ± 2.78	77.56 ± 3.52	0.193
Albumin, %	60.54 ± 1.40	60.26 ± 1.78	59.88 ± 2.65	59.72 ± 2.62	60.96 ± 2.40	60.08 ± 2.08	0.331
Yolk, %	29.51 ± 1.43	29.65 ± 1.80	30.07 ± 2.48	30.10 ± 2.07	28.81 ± 1.92	29.63 ± 1.39	0.142
Shell, %	9.95 ± 0.51	10.09 ± 0.61	10.05 ± 0.55	10.18 ± 0.86	10.23 ± 1.23	10.30 ± 1.04	0.695
Shell thickness, μm	263 ± 14.76^ab^	258 ± 16.26^b^	260 ± 20.23^b^	272 ± 13.59^a^	267 ± 16.05^ab^	271 ± 18.82^a^	0.008
Albumin index	8.49 ± 1.26	9.45 ± 1.68	8.62 ± 1.14	8.40 ± 1.84	9.60 ± 2.62	9.28 ± 2.70	0.090
Yolk index	41.33 ± 3.73^b^	42.89 ± 3.17^b^	43.02 ± 2.51^b^	45.74 ± 3.96^a^	47.09 ± 5.04^a^	45.62 ± 3.02^a^	0.001
Yolk color	6.26 ± 0.98	6.63 ± 0.74	6.63 ± 0.79	6.59 ± 0.84	6.56 ± 1.12	6.15 ± 1.17	0.263
Haugh unit score	84.64 ± 2.72	85.47 ± 3.25	84.21 ± 2.88	83.06 ± 4.50	84.86 ± 4.99	83.97 ± 5.40	0.362

^a,b^Means in the same row within each classification bearing different letters are significantly (p < 0.05) different

### Hematological and blood biochemical parameters

Table 4 shows the effect of dietary gamma-oryzanol supplementation on hematological and blood biochemical parameters in laying quails. Hb levels ranged from 26.95 g/dL to 28.93 g/dL and did not significantly differ between the groups (p > 0.05). Supplementation with gamma-oryzanol significantly increased the RBC count (p < 0.05), with the highest values at 0.4 g/kg. Quails in the 0 g/kg, 0.1 g/kg, and 0.2 g/kg diet groups exhibited significantly lower RBC counts than those in the 0.4 g/kg diet group. WBC count remained unaffected by gamma-oryzanol (p > 0.05), with values ranging from 3.29 to 4.00 × 10³ cells/μL. The TP, ALB, and GLOB concentrations were not significantly different among the treatments (p > 0.05). No significant changes in liver enzymes (AST and ALT) were observed across dietary treatments (p > 0.05). AST values ranged from 264.67 to 332.08 IU/L, whereas ALT levels were consistent at approximately 5.00 IU/L across all groups. In addition, there were no significant differences in creatinine and BUN among treatments.

**Table 4:** Hematology and blood biochemistry of laying quails fed with gamma-oryzanol supplementation (n = 6 birds/experimental group).

Item	Gamma oryzanol level (g/kg diet)						p-value

	0	0.1	0.2	0.3	0.4	0.5	
Hemoglobin (g/dL)	27.59 ± 2.30	28.93 8.63	26.95 ± 2.45	27.81 ± 3.07	28.60 ± 8.06	27.81 ± 2.81	0.820
RBCs (10^6^/mL)	3.40 ± 0.94^b^	3.69 ± 1.44^b^	3.72 ± 1.15^b^	3.72 ± 1.08^b^	4.64 ± 1.97^a^	4.10 ± 1.52^ab^	0.045
WBCs (10^6^/mL)	3.69 ± 2.08	3.50 ± 1.24	3.29 ± 1.64	4.00 ± 1.66	3.93 ± 1.60	4.00 ± 1.64	0.586
TP (g/dL)	4.96 ± 0.64	4.82 ± 0.55	4.93 ± 0.44	4.72 ± 0.57	4.96 ± 0.76	4.93 ± 0.72	0.957
ALB (g/dL)	1.66 ± 0.22	1.62 ± 0.18	1.64 ± 0.13	1.61 ± 0.16	1.63 ± 0.21	1.59 ± 0.30	0.987
GLOB (g/dL)	3.31 ± 0.43	3.20 ± 0.42	3.31 ± 0.34	3.12 ± 0.46	3.32 ± 0.60	3.34 ± 0.51	0.901
AST (IU/L)	294.83 ± 71.31	332.08 ± 134.04	295.00 ± 96.85	279.25 ± 91.46	264.67 ± 62.24	282.08 ± 114.62	0.659
ALT (IU/L)	5.00 ± 0.01	5.08 ± 0.29	5.00 ± 0.01	5.00 ± 0.01	5.00 ± 0.01	5.00 ± 0.01	0.425
Creatinine (mg/dL)	0.06 ± 0.01	0.07 ± 0.01	0.07 ± 0.02	0.08 ± 0.02	0.07 ± 0.01	0.08 ± 0.02	0.067
BUN (mg/dL)	1.58 ± 1.15	1.20 ± 0.25	1.39 ± 0.27	1.29 ± 0.20	1.24 ± 0.23	1.23 ± 0.33	0.661

RBCs = Total red blood cells, WBCs = Total white blood cells, TP = Total protein, ALB = Albumin, GLOB = Globulin, AST = Aspartate aminotransferase, ALT = Alanine aminotransferase. BUN = Blood urea nitrogen. ^a,b^Means in the same row within each classification bearing different letters are significantly (p < 0.05) different

### Ovarian index and number of ovarian follicles

Table 5 shows the effect of dietary gamma-oryzanol supplementation on ovarian function and follicle development in laying quails at 19 weeks. Ovarian index values (2.81–4.35) did not differ significantly among the treatment groups (p > 0.05). The number of follicles remained unaffected by gamma-oryzanol inclusion, with values ranging between 3.67 and 4.67. Supplementation did not significantly affect SYF and LWF counts (p > 0.05). Gamma-oryzanol had a significant effect on SWF numbers, with the highest count (66.00) observed at 0.5 g/kg diet, which was significantly higher than the control and 0.1 g/kg diet groups. The weight of the largest follicle (F1) ranged from 2.44 g to 3.27 g, while the second (F2), third (F3), and fourth (F4) follicles exhibited weights ranging from 1.26 g to 2.21 g, 0.26 g to 0.93 g, and 0.10 g to 0.33 g, respectively. F3 weight tended to increase in the 0.3 and 0.5 g/kg diet groups, although this trend was not statistically significant (p > 0.05). A significant increase in F3 follicle diameter was observed in the supplemented groups (p < 0.05), especially in the 0.3–0.5 g/kg diet groups. The F3 diameter was significantly larger in the 0.1, 0.3, 0.4, and 0.5 g/kg diet groups than in the control. In addition, F1 and F2 diameters exhibited a numerical increase in the 0.3 and 0.5 g/kg diet groups, though these differences were not statistically significant (p > 0.05).

**Table 5:** Ovarian index and follicle characteristics of laying quails fed with gamma-oryzanol supplementation (n = 6 birds/experimental group).

Item	Gamma oryzanol level (g/kg diet)						p-value

	0	1	2	3	4	5	
Ovarian index	3.56 ± 1.11	2.83 ± 0.20	2.81 ± 0.14	4.35 ± 1.95	3.15 ± 0.35	3.63 ± 0.24	0.374
Follicles number	3.67 ± 0.58	4.00 ± 1.00	4.00 ± 0.01	4.67 ± 1.15	4.00 ± 0.01	4.00 ± 0.01	0.618
SYF	0.67 ± 0.58	2.33 ± 3.21	0.33 ± 0.58	0.33 ± 0.58	0.67 ± 0.58	1.00 ± 1.00	0.571
LWF	15.67 ± 4.73	12.00 ± 4.36	16.33 ± 9.45	11.67 ± 6.66	15.33 ± 3.06	9.33 ± 1.53	0.601
SWF	17.67 ± 5.51^b^	15.67 ± 11.93^b^	37.67 ± 17.01^ab^	48.33 ± 20.84^a^	38.67 ± 21.57^ab^	66.00 ± 11.53^a^	0.017
Follicle weight (g)							
F1	2.86 ± 0.45	2.89 ± 0.67	2.44 ± 0.37	3.27 ± 0.84	2.94 ± 0.17	2.72 ± 0.61	0.630
F2	1.26 ± 0.83	1.58 ± 0.70	1.36 ± 0.38	2.21 ± 0.94	1.48 ± 0.42	1.55 ± 0.49	0.592
F3	0.26 ± 0.20^b^	0.60 ± 0.53^ab^	0.46 ± 0.18^ab^	0.93 ± 0.45^a^	0.67 ± 0.22^ab^	0.90 ± 0.21^a^	0.182
F4	0.10 ± 0.05	0.19 ± 0.13	0.10 ± 0.07	0.33 ± 0.42	0.14 ± 0.13	0.24 0.17	0.755
Follicle diameter (mm)							
F1	17.45 ± 1.19^ab^	17.78 ± 1.39^a^	16.03 ± 1.08^b^	18.07 ± 1.36^a^	17.34 ± 0.63^ab^	17.69 ± 1.35^a^	0.087
F2	13.54 ± 1.41^ab^	14.16 ± 2.41^ab^	13.18 ± 1.58^b^	15.43 ± 1.75^a^	14.15 ± 1.21^ab^	15.73 ± 1.66^a^	0.089
F3	7.24 ± 1.49^b^	9.70 ± 2.96^a^	8.93 ± 1.30^ab^	11.03 ± 1.23^a^	10.54 ± 0.84^a^	10.53 ± 1.83^a^	0.008
F4	5.64 ± 0.86	6.69 ± 1.59	5.49 ± 0.78	7.61 ± 2.15	6.10 ± 1.23	6.83 ± 2.65	0.339

SYF = Small yellow follicles, LWF = Large white follicles, SWF = Small white follicles, F = The numbers of primary follicles. ^a,b^Means in the same row within each classification bearing different letters are significantly (p < 0.05) different

## DISCUSSION

### Overall impact of gamma-oryzanol on laying quails

In this study, we evaluated the impact of dietary gamma-oryzanol on laying performance, egg quality, hematological and biochemical indices, and reproductive traits in Japanese quails. Gamma-oryzanol confers benefits on laying rate, feed efficiency, follicular development, and RBC production.

### Laying performance and feed efficiency

One of the most notable findings was the significant improvement in egg production at 0.2 g/kg diet supplementation, with numerical increases also observed at higher levels. This result is consistent with those of previous studies indicating that antioxidants can improve reproductive performance in poultry by reducing oxidative stress and enhancing ovarian function [14, 16]. Supplementation improved FCR and egg mass numerically, indicating enhanced nutrient utilization, although not statistically significant. These improvements are likely due to the antioxidant and anti-inflammatory effects of gamma-oryzanol on reproductive tissues [17].

### Eggshell and yolk quality enhancements

The improvements observed in eggshell quality at higher gamma-oryzanol levels suggest that this antioxidant enhances shell formation. The tendency toward increased shell thickness at higher doses (0.3–0.5 g/kg) suggests improved shell gland function and calcium use [18]. The finding of this study is consistent with the findings of Cao *et al*. [18], that antioxidants can bolster eggshell integrity by reducing oxidative damage in shell-forming tissues and improving calcium use under stress conditions.

The rise in the yolk index at higher inclusion levels indicates improved yolk membrane integrity, possibly due to lipid antioxidant protection. In other words, the antioxidative mechanisms of gamma-oryzanol help maintain the integrity of yolk lipids, resulting in a more robust yolk membrane and a higher yolk index in the egg.

In contrast, gamma-oryzanol supplementation did not significantly affect many general egg composition traits (albumen, yolk, and shell percentages) or certain aspects of internal quality (albumen index, Haugh units). These findings suggest that gamma-oryzanol selectively enhances structural traits (e.g., shell strength and yolk form) without affecting compositional parameters. The yolk color remained unchanged is expected, as yolk pigmentation is mainly driven by dietary carotenoids (such as xanthophylls) rather than antioxidant supplements [19]. Overall, the present findings indicate that gamma-oryzanol can be a beneficial additive for improving egg quality parameters (e.g., strengthening eggshells and enhancing yolk stability) without negatively impacting the normal composition of the egg. These enhancements are likely attributable to gamma-oryzanol’s antioxidant properties, which help reduce oxidative stress in laying quails.

### Shell thickness and mineral metabolism

Although most egg quality parameters, including egg weight, albumin content, and yolk color, were unaffected by gamma-oryzanol supplementation, shell thickness significantly improved at higher inclusion levels (0.3–0.5 g/kg). Similar findings have been reported in studies on other natural antioxidants, which suggest that oxidative stress can negatively affect eggshell mineralization, and antioxidant supplementation helps in maintaining shell strength [1]. The improvement in shell thickness suggests that gamma-oryzanol may play a role in calcium metabolism and shell formation, possibly by enhancing bone metabolism and reducing oxidative stress in shell gland tissues [20]. Improved shell thickness reduces egg breakage and improves overall egg quality, which is crucial for commercial egg production.

### Hematological response and safety

A significant increase in RBC count, especially at 0.4 g/kg diet, indicates stimulated erythropoiesis, likely due to enhanced red cell integrity under oxidative control. This is in agreement with a previous study of Sahin *et al*. [21], showing that dietary antioxidants can promote erythropoiesis by reducing oxidative stress-induced damage to erythrocytes and improving iron metabolism. This may be due to its antioxidative effects, which can enhance cellular integrity and promote RBC survival.

Other blood parameters, including Hb, WBC count, liver enzyme levels, and kidney markers, remained stable, indicating no systemic toxicity. Similarly, liver function enzymes (AST and ALT), renal function markers (creatinine and BUN), and total protein levels were unaffected, indicating that gamma-oryzanol does not exert any hepatotoxic or nephrotoxic effects. This finding supports the safety of gamma-oryzanol supplementation in laying quails.

### Follicular development and ovarian function

Gamma-oryzanol enhanced follicular development, as evidenced by a significant increase in the number of SWFs and F3 follicle diameter at higher doses. These findings are consistent with previous research suggesting that antioxidants play a crucial role in follicular recruitment and ovarian function [22]. Enhanced folliculogenesis likely contributes to higher egg production, reflecting improved ovarian responsiveness [23].

### Mechanisms of reproductive enhancement

The reproductive and performance benefits of gamma-oryzanol are largely attributed to its antioxidative actions in ovarian and metabolic tissues. Since oxidative stress impairs fertility and ovarian function, antioxidants, such as gamma-oryzanol, play a key role in preserving reproductive health [24]. Gamma-oryzanol, a natural antioxidant, likely helps to mitigate oxidative damage, leading to improved ovarian function, feed efficiency, and egg production.

In addition, its anti-inflammatory effects may contribute to improved nutrient utilization and overall metabolic homeostasis, which is reflected in the improved laying performance and follicular development observed in this study. Gamma-oryzanol could serve as a functional feed additive for improving laying performance and egg quality in commercial poultry production.

### Implications for fertility and embryonic health

The antioxidative action of gamma-oryzanol is thought to play a key role in supporting the reproductive function of birds. Oxidative stress is known to impair fertility and embryo development; therefore, the inclusion of antioxidants in breeder diets can protect reproductive cells (ova and sperm) from damage [25, 26]. Gamma-oryzanol, in particular, has demonstrated anti-apoptotic effects in oxidatively stressed cells by suppressing mitochondrial damage mediated by reactive oxygen species [26].

In practical terms, feeding gamma-oryzanol-rich supplements to breeder birds can result in better reproductive outcomes. Quail-specific studies have shown that adding RBO (rich in gamma-oryzanol) to quail diets significantly increased fertility rates and hatchability of eggs [6]. This improvement likely results from enhanced antioxidant protection of ovarian follicles and embryos.

Antioxidants from the maternal diet, including gamma-oryzanol, may be deposited in yolks, enhancing embryonic oxidative defense [27]. By enriching the maternal diet with gamma-oryzanol, quail hens may transfer protective compounds to their follicles and eggs, thereby boosting embryo viability and chick quality. In addition, gamma-oryzanol’s lipid-modulating effects might optimize the availability of cholesterol for steroid hormone synthesis in the ovaries, indirectly supporting follicle growth and ovulation. These mechanisms underscore the potential of gamma-oryzanol in improving follicular development, hormone regulation, and overall reproductive efficiency in laying quails.

## CONCLUSION

This study demonstrated that dietary supplementation with gamma-oryzanol significantly enhances laying performance, egg quality, RBC production, and follicular development in Japanese quails without inducing any adverse hematological or biochemical effects. Specifically, supplementation at 0.2 g/kg diet resulted in the highest egg production (98.69%), while 0.3–0.5 g/kg levels improved shell thickness and yolk index. A significant increase in RBC count was observed at 0.4 g/kg, indicating hematological benefits. In addition, gamma-oryzanol increased the number of SWF and F3 follicle diameter, reflecting improved follicular recruitment and ovarian function.

From a practical standpoint, gamma-oryzanol presents a viable natural feed additive to enhance both productivity and reproductive efficiency in commercial quail farming. Its antioxidant and anti-inflammatory properties contribute to improved egg quality and systemic physiological stability, potentially reducing reliance on synthetic additives or hormonal enhancers. The safety profile observed further supports its long-term dietary use in avian species.

One of the key strengths of this study lies in its comprehensive evaluation – encompassing laying performance, internal and external egg quality traits, blood health, and follicular parameters – thereby offering a holistic understanding of gamma-oryzanol’s effects. In addition, the inclusion of multiple supplementation levels allows for identification of a potentially optimal dosing range (0.2–0.4 g/kg).

However, the study also presents limitations. The experimental duration was relatively short (7 weeks), which may not fully capture long-term reproductive or metabolic shifts. Hormonal profiles and oxidative stress biomarkers were not assessed, limiting mechanistic interpretation. Furthermore, fertility and hatchability data were not included, which are critical for evaluating outcomes in breeder operations.

For future research, longer-term feeding trials are warranted to evaluate the sustainability of benefits and the cumulative physiological effects. Investigations into endocrine regulation, gene expression related to folliculogenesis and steroidogenesis, and embryo viability should be prioritized. Additionally, cost-benefit analyses of gamma-oryzanol supplementation in large-scale production systems would enhance its applicability in the industry.

In conclusion, gamma-oryzanol is a promising functional feed additive for laying quails, capable of improving productivity, reproductive health, and product quality in a safe and natural manner. With further validation and optimization, it holds strong potential for adoption in sustainable poultry nutrition strategies.

## AUTHORS’ CONTRIBUTIONS

KW, WI, and AW: Designed and conducted the study. KW, WI, AW, AK, and CL: Analyzed and interpreted the data. KW WI, AW, PT, AK, and CL: Drafted and edited the manuscript. PT, AW, and KW: Conceptualized the study, methodology, and drafted the manuscript. All authors have read and approved the final manuscript.
